# Effect of Intermittent Subglottic Irrigation with 5% NaCl on the Prevention of Ventilator Associated Pneumonia in Critically Ill Patients

**Published:** 2019-02

**Authors:** Taraneh Naghibi, Zahra Akbari, Somayae Abdollahi Sabet, Faramarz Dobakhti

**Affiliations:** 1 Department of Anesthesiology and Critical Care Medicine, Mosavi Educational Hospital, School of Medicine, Zanjan University of Medical Science, Zanjan, Iran,; 2 Department of Community Medicine, School of Medicine, Social Determinants of Health Research Center, Zanjan University of Medical Sciences, Zanjan, Iran,; 3 School of Pharmacy, Zanjan University of Medical Science, Zanjan, Iran

**Keywords:** 5% Sodium hypertonic, Subglottic irrigate, Ventilator-associated pneumonia

## Abstract

**Background:**

Ventilator-Associated Pneumonia (VAP) is one of the common causes of mortality and morbidity. Subglottic secretion suction decreases the incidence of VAP. In this study, the effect of 5% sodium chloride (NaCl) in subglottic area in addition to secretion suction in VAP prevention was investigated in patients who were admitted to the intensive care unit.

**Materials and Methods:**

All patients were intubated by an intubation tube with subglottic suction. In the intervention group, subglottic area was washed with 10 ml of **5%** sodium chloride solution, and in the control group the subglottic area was washed with 10 ml distilled water. Patients were monitored for a maximum of two weeks, and the incidence of VAP was monitored by a Clinical Pulmonary Infection Score (CPIS).

**Results:**

There was no significant difference between the two groups in terms of age and sex. Four (27%) patients in the intervention group, and 7 (37%) in control group were diagnosed with VAP, which was not statistically significant between the two groups (P=0.225). The duration of hospitalization, duration of intubation and mortality did not show any significant difference between the two groups.

**Conclusion:**

It was expected that in this study the rate of VAP became significantly lower with the intervention of using antimicrobial solution in addition to suction. Although the rate decreased clinically, it was not statistically significant, which may be due to the low number of patients.

## INTRODUCTION

Ventilator-Associated Pneumonia (VAP) is a serious and common disorder in the Intensive Care Unit (ICU). The incidence of VAP is 9–27% in critically ill patients who are under mechanical ventilation. VAP remains a major contributor to morbidity and mortality in intubated patients (1). It is associated with dangerous complications in this group of patients. These complications include prolonged ventilation support, ICU and hospital length of stay, and increased healthcare cost (2, 3). Pathogenic micro-organisms that colonize the oropharyngeal region are the most important cause of VAP (4). One of the important ways which predisposes patients to VAP is micro aspiration of subglottic secretions that are above the cuff of endotracheal tube. Significant changes in liquid aspiration are due to materials and design of the endotracheal tube cuff (5). After intubation, pathogenic bacteria colonize the oropharynx surfaces (6, 7) and secretions rapidly accumulate above the cuff (8). By lining the cuff these secretions enter the lungs and create infection. For this reason preventive strategies for VAP are based on the colonization and aspiration modification. One of these strategies includes subglottic secretion draining. Based on these reasons a kind of endotracheal tube has been made through which secretions from the subglottic region can be suctioned. Although studies have shown that subglottic secretion suctioning decreases the incidence of VAP, it is still the most common cause of death in ICU (9). Therefore, use of an antimicrobial agent in the subglottic region may have a better effect on VAP reduction than suctioning the secretion alone.

Sodium chloride (NaCl) by increasing osmotic pressure and decreasing water activity around the microbes stops microbial growth. Although the salt environment is suitable for some bacteria, such as *Staphylococcus saprophyticus*, these micro-organisms cannot live in high salt concentrations. Other bacteria such as Escherichia coli have the best growth in salt-free environments and salt can severely limit its growth (10).

Many studies have shown the effect of hypertonic sodium chloride nebulizer on the improvement of cystic fibrosis patients (11). Previous studies have shown that hypertonic sodium chloride reduces the activity of *Pseudomonas aeruginosa* and improves patients who suffer from cystic fibrosis. It can also be effective in improving other pulmonary patients (12). It has been shown that hypertonic sodium chloride has a static and a bactericidal effect. Also, when is used topically on wounds can prevent the spread of infection (13). Oguzhan et al. have stated that hypertonic sodium chloride reduces the catheter-dependent infection. They have shown that hypertonic sodium chloride has bactericidal effect on *Escherichia coli* and Pseudomonas, but has a bacteriostatic effect on *Staphylococcus aureus* and *Staphylococcus epidermidis* (14). According to the bactericidal effect of hypertonic sodium, a comparative study was designed to indicate the effect of 5% sodium chloride injection in the subglottic region in prevention of VAP. It should be noted that as this was the first study in this regard; therefore, it was designed as a pilot study.

## MATERIALS AND METHODS

### Study design

This study is a randomized, double-blind, placebo-controlled trial. After approval by the Ethics Committee (approval number: IR.ZUMS.REC.1396.207), 30 patients admitted to the ICU between Feb 2016 and Oct 2017, were enrolled in this study. The investigation was conducted in a 21-bed department of the ICU in our tertiary health care institution in a university educational hospital.

The trial is registered with the Iranian Registry of Clinical Trials (IRCT20101211005363N10). Consent form was obtained from the patient's relatives.

### Patient population

A convenience sample was taken from all surgical patients who were admitted to the ICU within 24 hours of intubation. Other inclusion criteria were as follows: patients age between 20 and 50 years, Acute Physiology and Chronic Health (APACHE) II score between 15–25, patient’s family satisfaction to participate in the research, not taking antibiotics, have no jejunostomy, not on intravenous feeding, have no tracheostomy, patients with teeth, and no evidence of pneumonia. Patients were excluded from analysis for the following reasons: patients did not survive for at least one week after admission, patients who were extubated before one week after admission and failure to start the gavage in the first 48 hours after intubation.

### Randomization and blinding

Patients who met the inclusion criteria were randomly allocated (1:1) to sodium chloride or placebo group according to a randomization code listed by a computer program. In this study, patients were not aware of the treatment group due to the decreased level of consciousness. The researcher, who studied the variables, did not know the group of study, and washing of subglottic was done by a nurse.

### Clinical Assessments

Patient's demographic data included age and sex, and APACHE score II was recorded at the time of admission. All patients were intubated by suction tube (Tra-suction). Every 8 hours, the tube cuff was adjusted between 25 and 30 cm Hg. Subglottic secretion of patients was aspirated by a lumen which was used for this purpose in the Tra-suction tube. Afterward, in the sodium chloride group, 10 ml of 5% saline solution and in the placebo group 10 ml distilled water was entered into the subglottic region through the suction lumen. The solutions were suctioned after 5 minutes. Then the tube cuff was adjusted to 20 to 25 cm Hg again. Patients were monitored for up to two weeks during mechanical ventilation. The incidence of VAP was measured by the Clinical Pulmonary Infection Score (CPIS), which was measured every three days.

### Outcome

Primary outcome was the incidence of VAP during mechanical ventilation up to two weeks. Secondary outcomes were duration of mechanical ventilation, length of ICU stay and mortality during hospitalization in ICU.

### Statistical analysis

In describing the quantitative variables due to the small number of samples which were not normally distributed, median (med) and Interquantile Range (IQR) were reported; moreover, qualitative variables were described by percentages. The quantitative data were compared through Mann-Whitney U test, while comparing qualitative data, the Exact test was used. Statistical significance level was set at 0.05.

## RESULTS

### Patient characteristics

Recent investigation was a pilot study. Thirty patients were enrolled in this randomized, double- blind, placebo-controlled trial. Fifteen patients were enrolled in each group. Patient characteristics were comparable in age, sex, APACHE II Score, underlying disease and cause of hospitalization upon admission (Table 1).

**Table 1.** Baseline characteristics of patients

		**Sodium chloride group (n = 15)**	**Placebo group (n = 15)**	**P-value**
**Age years, med ± IQR**		38±14	45.57±14.53	P=0.983
**Male gender, no. (%)**		10(66.7)	9(60)	P=1.000
**APACHE II, med ± IQR**		20±4	19±2	P=0.247
**Underlying disease**	**+**	60	27	P=0.139
	**−**	40	73	

There was no statistically significant difference between groups (P > 0.05).

APACHE II: Acute physiology and chronic health

med: median IQR: interquantile range

### Outcome

Incidence of VAP and duration of mechanical ventilation days, length of ICU stay and mortality during hospitalization in ICU were not significantly different between groups (Table 2).

**Table 2.** Outcome of the included patients

**Outcome**	**Sodium chloride group (n=15)**	**Placebo group (n=15)**	**P-value**
**Incidence of VAP, no. (%)**	4(26.6)	7(47)	0.2
**Duration of mechanical ventilation, days (med ± IQR)**	10±3	12±4	0.314
**Length of ICU stay, days (med ± IQR)**	21±14	23±11	0.803
**ICU mortality, no.(%)**	4(26.7)	3(20)	1

Statistically significant difference between groups (P < 0.05).

med: median IQR: interquantile range

VAP: Ventilator-associated pneumonia

ICU: intensive care unit

## DISCUSSION

Based on our findings, this is the first study to examine the effect of subglottic irrigation with 5%NaCl on the prevention of VAP. For this reason, the Ethics Committee only allowed to conduct the investigation as a pilot study and on limited patients.

The incidence of VAP in the intervention group was reduced in this study; however this decline was not statistically significant. Secondary outcomes including duration of mechanical ventilation, duration of hospitalization in ICU and mortality, were also analyzed. There were no statistically significant differences in any of these results. Gopal et al. have investigated a study on 240 patients who were admitted to the ICU. They have reported that Venner-PneuX ETT could reduce the incidence of VAP in this population of patients (P=0.03). They did not find significant difference in the mortality rate and ICU stay between the two groups (P=0.2) (15).

In a study conducted by Walaszek et al. in 2017, the effectiveness of subglottic secretion suction in the prevention of VAP was evaluated. The results showed that subglottic secretion suction significantly reduced the incidence of VAP (16). In all of above studies incidence of VAP is reduced by suction of subglottic secretion. In the present study, it was expected that the incidence of VAP after using antimicrobial solution, in addition to suction be significantly lower than suction of secretions alone. The incidence of VAP clinically decreased, although it was not statistically significant.

Ayhan et al. in a multimethod study have shown that normal saline instillation in the tracheal tube significantly decreases the oxygenation, so they have recommended that this procedure should not be used (17). For prevention of this complication, in the present study saline solution was instilled in subglottic area, the main place of micro-aspiration that leads to VAP.

Caruso et al. have shown that instillation of saline prior to tracheal suction due to stimulating a cough and thinning secretions which might decrease the risk of VAP (18). However, a number of studies have shown that it can increase bacterial dislodgement and can lead to its migrating into the lower airways. We increased the cuff pressure during saline instillation to prevent bacterial dislodgement, and a hyper-tonic saline was used above the cuff. However, there is a possibility that micro-aspiration could cause these inappropriate results in the recent study.

Meanwhile, as regards to low power of the study, the limited number of patients can lead to these results.

Limitations of our study were: the study was carried out in a single institute, and had a small sample size. These limitations have affected the strength of our study, but the method of analysis and its interpretation are appropriate.

## CONCLUSION

It can be concluded that subglottic irrigation with 5%NaCl in addition to suction, can clinically reduce the incidence of VAP, although this reduction is not significant.
